# The Multifaceted Effects of Alpha1-Antitrypsin on Neutrophil Functions

**DOI:** 10.3389/fphar.2018.00341

**Published:** 2018-04-17

**Authors:** Sabina Janciauskiene, Sabine Wrenger, Stephan Immenschuh, Beata Olejnicka, Timm Greulich, Tobias Welte, Joanna Chorostowska-Wynimko

**Affiliations:** ^1^Department of Respiratory Medicine, Biomedical Research in Endstage and Obstructive Lung Disease Hannover (BREATH), Member of the German Center for Lung Research (DZL), Hannover Medical School, Hannover, Germany; ^2^Department of Genetics and Clinical Immunology, National Institute of Tuberculosis and Lung Diseases, Warsaw, Poland; ^3^Institute for Transfusion Medicine, Hannover Medical School, Hannover, Germany; ^4^Department of Medicine, Trelleborg Hospital, Trelleborg, Sweden; ^5^Department of Medicine, Pulmonary and Critical Care Medicine, Member of the German Center for Lung Research (DZL), University Hospital of Giessen and Marburg, University of Marburg, Marburg, Germany

**Keywords:** neutrophil granulocyte, Alpha1-Antitrypsin, acute phase protein, innate immunity, inflammation, proteases, cytokines, neutrophil degranulation

## Abstract

Neutrophils are the predominant immune cells in human blood possessing heterogeneity, plasticity and functional diversity. The activation and recruitment of neutrophils into inflamed tissue in response to stimuli are tightly regulated processes. Alpha1-Antitrypsin (AAT), an acute phase protein, is one of the potent regulators of neutrophil activation via both -protease inhibitory and non-inhibitory functions. This review summarizes our current understanding of the effects of AAT on neutrophils, illustrating the interplay between AAT and the key effector functions of neutrophils.

## Introduction

Neutrophils are the most abundant leukocyte type in the human circulation, generated in the bone marrow at a rate of 10^11^ a day whereas during bacterial infection can increase to 10^12^ a day. Studies using mice models show that the bone marrow may not be the only source of neutrophils. For example, haematopoietic progenitors present in the circulation of mice can accumulate in infected tissues where they differentiate into mature and functional neutrophils (Granick et al., 2013).

Historically, human neutrophils have been viewed as short-lived cells representing the first line of defense in response to invading pathogens. However, there is emerging evidence that neutrophils are involved not only in the killing of extracellular pathogens, but also contribute to the immune responses through cross talk with other immune cells, such as lymphocytes, dendritic cells and natural killer cells. Neutrophils express and secrete different substances, and express a large number of cell surface molecules to interact with other cells. Indeed, recent studies, which are summarized in depth elsewhere, are changing our perception on role of neutrophils in host defense (Mayadas et al., 2014; Soehnlein et al., 2017).

Neutrophil activation, mobilization and accumulation are tightly regulated events by a variety of both pathogen and host factors. Among these, acute-phase proteins, whose serum levels change by >25% during inflammation (Gabay and Kushner, 1999), are important modulators of innate immune cells, such as neutrophils (Sander et al., 2010). In this review, we focus on Alpha1-Antitrypsin (AAT) that plays a role in controlling neutrophil activation and neutrophil-initiated processes.

**FIGURE 1:**
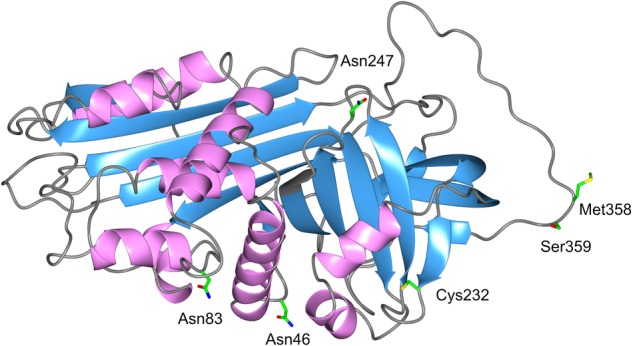
3D structure of AAT. The AAT protein is a 394-amino acid peptide with one free cysteine residue (marked in the structure) and three asparagine-linked carbohydrate side-chains at positions 46, 83, and 247. The AAT polypeptide chain is arranged into structural elements consisting of three beta-sheets (blue color) and nine alpha-helices (purple color), each formed by the first 150 residues. A reactive center loop presents the key Met358–Ser359 (P1-P1′) residues for the cleavage by the target proteases. This active site designated “P1 residue” is responsible for the anti-protease activity and specificity of the inhibitor. Side chains of amino acids of interest are colored as carbon (green), oxygen (red), nitrogen (blue), and sulfur (yellow).

Alpha1-Antitrypsin, also referred to as α_1_-proteinase inhibitor or serpin A1, is an acute phase protein. It is the most abundant serine proteinase inhibitor in human plasma, and is encoded by the SERPINA1 gene (located on the long arm of the 14th chromosome, 14q32.1). Crystallographic analysis revealed that AAT is a single polypeptide chain glycoprotein containing one free cysteine residue and three asparagine-linked carbohydrate side-chains (Mega et al., 1980; Carrell et al., 1981; Long et al., 1984) (**Figure 1**). A cysteine residue can be easily oxidized to form a dimer with a disulphide bridge between two cysteines (Na et al., 2015). It is noteworthy that covalent homodimers mediated by inter-Cys232 bonding are not seen for the wild type variant of AAT (so called “M” AAT) but are observed in Z and other polymerising AAT variants where protein folding is aberrant (Ronzoni et al., 2016).

**FIGURE 2:**
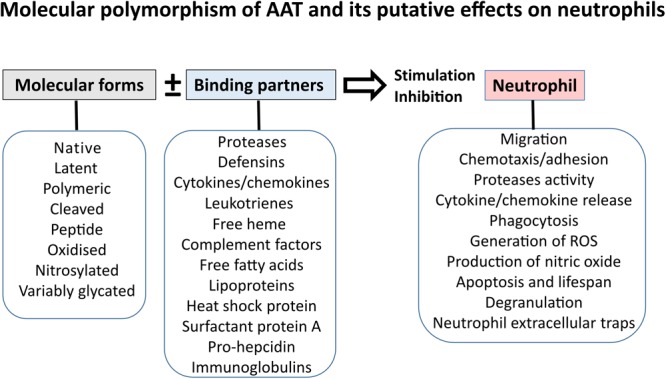
Effects of AAT on neutrophil functions might depend on molecular form and binding partner of AAT.

Alpha1-Antitrypsin exhibits a potent inhibitory capacity against neutrophil serine proteases, particularly neutrophil elastase (NE), which is considered its main physiological function. In fact, people with inherited AAT deficiency (AATD) have reduced levels of circulating AAT, which is strongly linked to an increased risk of developing early-onset pulmonary emphysema (Laurell and Eriksson, 1963; Hutchison, 1973), a pathology, at least in part, characterized by the protease-antiprotease imbalance. To correct this imbalance, preparations of purified AAT protein from human plasma are used as a therapy. Recently, preparations of AAT have become a ‘hot-topic’ in therapeutic strategies for other inflammatory diseases. This is because AAT appears to exert quick immune modulatory functions other than its classic anti-neutrophil protease activity. Furthermore, some of the AAT functions are based on its particular molecular form. AAT can be oxidized, cleaved, self-assembled or complexed with lipids and other molecules, which influences its biological activity (Janciauskiene and Welte, 2016). These findings also lead to the hypothesis that AAT can perform divergent roles in the regulation of neutrophil functions and neutrophilic inflammation depending on its quantitative and qualitative characteristics (**Figure 2**).

## Neutrophils Are the Source of AAT

Liver cells synthesize and release most of the circulating AAT in the blood (normal levels vary between 0.9 and 2 g/L). Likewise, epithelial cells, pulmonary alveolar cells, tissue macrophages, blood monocytes and granulocytes also contribute to the pool of circulating AAT. Different studies have demonstrated that AAT is a component of eosinophil (Johansson et al., 2001) and neutrophil granules (Mason et al., 1991). The granule proteins of circulating neutrophils are produced during maturation of neutrophil precursors in the bone marrow. The mRNA for AAT is found at the promyelocyte stage of differentiation and is highly upregulated in mature polymorphonuclear cells (Clemmensen et al., 2011). We analyzed the responses of freshly isolated human blood neutrophils to lipopolysaccharide (LPS) and unexpectedly found that LPS not only upregulates expression of full-length AAT protein but also the expression of short transcripts of the SERPINA1 gene (Matamala et al., 2017). In parallel, liquid chromatography-mass spectrometry analysis identified the presence of C-terminal peptides of AAT in supernatants from LPS-activated neutrophils. Interestingly, a recent study found that the specific C-terminal fragment of AAT expresses immunomodulatory functions, particularly on human neutrophils during severe sepsis (Blaurock et al., 2016). In general, peptides of AAT are thought to be the result of cleavage of AAT by non-specific proteases, like neutrophil collagenases and gelatinases, among others (Michaelis et al., 1992; Pei et al., 1994; Zhang et al., 1994; Liu et al., 2000). However, the emergence of new findings re-opens the question whether free peptide(s) of AAT are exclusive products of AAT cleavage, or can also be *de novo* synthesized by neutrophils or other cells.

Previous studies have found that neutrophil AAT is localized within primary granules (Mason et al., 1991), secretory vesicles (Paakko et al., 1996), or all granule subtypes (Clemmensen et al., 2011). Clemmensen et al. (2011) pointed out that AAT stored in azurophil granules is not released during the activation of neutrophils and furthermore does not seem to form complexes with the NE, proteinase 3 (PR3), or cathepsin G (CG) that are also present in the same azurophil granules. On the other hand, findings by Bergin et al. suggest that most of the neutrophil-associated AAT is localized to the cell membrane within lipid rafts (Bergin et al., 2010). Similarly, we found that exogenous AAT added to adherent human peripheral blood mononuclear cells is localized in lipid rafts together with flotillins, the components of lipid-rafts (Subramaniyam et al., 2010).

Taken together, existing data imply that neutrophils might represent a local source of AAT. Moreover, they may potentially be a source of shorter transcripts of AAT with as yet unidentified functions. Pathways that govern the basal level of AAT synthesis, storage, and trafficking in human neutrophils are still poorly understood.

## AAT Is an Inhibitor of Neutrophil Serine Proteases

Neutrophil serine proteases, NE, PR3, and CG are highly active proteolytic enzymes that are formed during the promyelocytic phase of neutrophil maturation and mainly stored in azurophilic granules. Neutrophil activation by cytokines like tumor necrosis factor-α (TNF-α), chemoattractants (platelet-activating factor or interleukin [IL]-8), or bacterial LPS, leads to a rapid granule translocation to the cell surface and extracellular secretion of NE, PR3, and CG (Owen and Campbell, 1999). A fraction of secreted proteases are also detected at the surface of activated neutrophils (Campbell et al., 2000).

Released serine proteases usually work synergistically. For example, data from animal models imply that NE is required for the clearance of certain gram-negative bacteria (Belaaouaj et al., 1998), CG is essential for resistance against infection with *Staphylococcus aureus* (Reeves et al., 2002), and both are effective against fungal infections (Tkalcevic et al., 2000). Likewise, NE, PR3 and CG mediate the release of the chemokine, IL-8, by engaging different receptors such as toll-like receptors (TLRs), protease-activated receptors (PARs), and integrins (Kessenbrock et al., 2011). All three proteases can process cytokines of the IL-1 superfamily, such as IL-1β, IL-18, and IL-33, into biologically active forms (Afonina et al., 2015). In support of this, mice with a triple deficiency of NE, PR3, and CG were better protected against smoke-induced emphysema than single elastase-deficient knockout mice (Guyot et al., 2014). A substantial release of neutrophil serine proteases into the extracellular space may be detrimental to the entire organism if not opposed by endogenous inhibitors such as AAT.

Alpha1-Antitrypsin is a well-recognized inhibitor of human neutrophil serine proteases. The second-order constants of association of AAT with NE, PR3, and CG are 6.5 × 10^7^, 8.1 × 10^6^, and 4.1 × 10^5^ M^-1^ s^-1^, respectively (Beatty et al., 1980; Rao et al., 1991). Crystallographic studies have revealed that the binding of neutrophil serine proteases to AAT cleaves the reactive center loop of AAT, which destroys both the protease and AAT. Cleavage of the reactive center loop of AAT results in the complex formation, in which the protease is flipped to the opposite end of the AAT molecule (Elliott et al., 1996; Zhou et al., 2001; Dementiev et al., 2003).

The inhibitory mechanism of AAT also involves its several methionine residues, which can be easily oxidized (Johnson and Travis, 1979). Indeed, when AAT is oxidized (especially Met-358 in the reactive loop), its inhibitory capacity is diminished or lost (Taggart et al., 2000). Interestingly, early studies revealed that oxidation of Met-358 to methionine sulfoxide affects AAT-protease complex formation, but not the interaction between AAT and protease, since replacement of the methionine with valine does not interfere with the inhibitory activity of AAT (Rosenberg et al., 1984). The oxidized AAT is considered as a potential marker of neutrophil activation associated with secretion of myeloperoxidase, a peroxidase enzyme that is a major component of neutrophil azurophilic granules (Ueda et al., 2002).

The interactions of AAT with DNA, heparin and other glycosaminoglycans found at inflammatory sites can also affect the association of AAT with serine proteases (Frommherz and Bieth, 1991; Frommherz et al., 1991; Belorgey and Bieth, 1998; Ying and Simon, 2000). For example, DNA and heparin decrease the rate constant of association between CG and AAT (Ermolieff et al., 1994; Duranton et al., 2000), whereas heparin prevents AAT-NE conversion into a covalently stable complex (Faller et al., 1993).

## AAT Interaction With Ne Versus PR3

The anti-protease functions of AAT are often extrapolated from the available data on NE, because AAT is considered as the most potent inhibitor of NE. When NE and PR3 are simultaneously present, AAT shows a preference for NE inhibition over PR3 (Korkmaz et al., 2005). Because AAT forms complex with NE at a faster rate, PR3 remains uninhibited for a longer period and may act more profoundly than NE (Sinden et al., 2015). PR3 can cleave the pro-form of TNF-α (Robache-Gallea et al., 1995) and the full-length pro-IL-8, liberating TNF and the most active form of IL-8 (Padrines et al., 1994). Moreover, it has been suggested that PR3 has a greater role than NE in IL-1β processing and secretion (Coeshott et al., 1999). Neutrophils are a major source of IL-1β (Aggarwal et al., 2016), and neutrophil-derived IL-1β mediates further neutrophil recruitment and activation. IL-1β is a highly inflammatory cytokine implicated in various pathological conditions.

Unlike NE and CG, PR3 is constitutively present in the membranes of freshly isolated neutrophils (Csernok et al., 1990; Halbwachs-Mecarelli et al., 1995). Several studies support the hydrophobic nature of the PR3-membrane interaction (Goldmann et al., 1999; Korkmaz et al., 2004, 2008; Hajjar et al., 2008; Broemstrup and Reuter, 2010) and show PR3 co-localisation with integrin CD11b/CD18 (beta-2 integrin), the Fcγ receptor FcγRIIIb and the p22phox subunit of cytochrome b558 (Hajjar et al., 2010). Notably, PR3 localizes in the lipid raft domains of neutrophil membranes (David et al., 2005; Fridlich et al., 2006). The same positioning was also described for AAT (Subramaniyam et al., 2010). According to previous studies, AAT is able to inhibit neutrophil membrane-bound NE and PR3 (Korkmaz et al., 2005, 2009). Moreover, Rooney et al. (Rooney et al., 2001) demonstrated that AAT prevents anti-PR3 IgG binding to the PR3 on the surface of the neutrophil, which in turn prevents PR3–FcγRIIa cross linkage and cell activation. In support of this, different studies have also shown that AAT inhibits IL-8, TNF-α and IL-1β release from activated neutrophils (Ralston et al., 1997; Nita et al., 2005; Ehlers, 2014; Aggarwal et al., 2016). Therefore, the putative interaction between AAT and PR3 within lipid-rafts might be as important as that between AAT and the soluble form of PR3 (Goldmann et al., 1999).

## AAT as an Inhibitor of Neutrophil Non-Serine Proteases

The modulation of neutrophil functions by AAT was mainly attributed to its anti-neutrophil serine protease activity. However, studies have also shown that AAT directly inhibits the activity of caspase-3, an intracellular cysteine protease that plays an essential role in cellular apoptosis (Petrache et al., 2006). Caspase-3 activation during neutrophil death is well documented (Luo and Loison, 2008). Recent findings provide evidence that cleavage and activation of pro-caspase-3 results from the release of PR3 from granules into the cytosol. PR3-mediated caspase-3 activation seems to play a critical role in a programmed neutrophil death (Loison et al., 2014).

The cytosolic activity of PR3 can be neutralized by SERPINB1 (also known as leukocyte elastase inhibitor, LEI) (Gettins, 2002), but there is also evidence that AAT plays a role in preventing cellular apoptosis. For instance, neutrophils isolated from severely injured people show delayed apoptosis (Paunel-Gorgulu et al., 2012). The same authors recently found that these neutrophils have high mRNA expression of AAT, and that AAT is retained in the cells (Sarabhai et al., 2017). Other authors have reported that AAT inhibits ADAM-17 activity (also called tumor necrosis factor-α–converting enzyme: TACE) (Bergin et al., 2010), which is necessary for activation of the death receptor pathway through caspase-8 cleavage, and that AAT therapy diminishes neutrophil apoptosis by reducing ADAM-17 activity (Hurley et al., 2014). Whether neutrophil resistance to apoptosis relates to the ability of AAT to inhibit caspase-3 and/or PR3 and ADAM-17, warrants further investigation.

Alpha1-Antitrypsin is also thought to be involved in the inhibition of gelatinase B (MMP-9) in neutrophils. Neutrophils have been identified as the predominant source of MMP-9 (Bradley et al., 2012). MMP-9 is formed in the later stages of neutrophil maturation and contributes to neutrophil extravasation and stem cell mobilization via the degradation of basement membrane collagens (Opdenakker et al., 1998). AAT may be an indirect physiological inhibitor of MMP-9 because it inactivates elastase, an activator of MMP-9 (Ferry et al., 1997). On the other hand, AAT is considered as a key protein substrate of MMP-9 (Liu et al., 2000). Hence, a vicious cycle may be involved whereby increased MMP-9 activity inactivates AAT, leading to diminished anti-NE capacity. In support of this hypothesis, excessive neutrophil infiltration into the lung correlates with increased MMP-9 production (Narasaraju et al., 2011), and MMP-9 was suggested as a biomarker to predict decline of lung function in patients with AATD (Omachi et al., 2011). By contrast, serum MMP-9 levels seem to be lower in AATD patients receiving AAT therapy than in those without it (Koepke et al., 2015).

There is additional evidence that AAT may offer substantial protection against neutrophilic inflammation because of its ability to inhibit calpain I (Al-Omari et al., 2011). In contrast to many other cell types, the inhibition of neutrophil μ-calpain (calpain I) activity promotes rapid neutrophil polarization, spreading and random migration. For example, Lokuta et al. (2003) demonstrated that calpain I inhibitors promote random neutrophil migration and polarization but decrease directional migration toward chemotactic stimuli. Calpain inhibitors have also been found to induce intracellular Ca^2+^ (Wiemer et al., 2010), and to activate Rho GTPases (Price et al., 2003; Pankov et al., 2005). Similarly, a transient increase in Ca^2+^ after neutrophil exposure to AAT occurred in parallel with rapid and transient activation of Rac1, Cdc42, and ERK1/2. Calpain I inhibition-mediated neutrophil polarization seems to be dependent on the Rho GTPase and Rac-signaling pathways, e.g., the ERK1/2 pathway (Katsube et al., 2008). In support for this, AAT was found to inhibit chemotaxis of activated neutrophils *in vitro* (Janciauskiene et al., 2004).

Several studies have shown that calpain may be activated in the cytosol and subsequently translocated to the membrane after interaction with phospholipids (Pontremoli et al., 1985; Arthur and Crawford, 1996; Al-Omari et al., 2011). Because AAT can localize in lipid rafts (Bergin et al., 2010; Subramaniyam et al., 2010), it is plausible that AAT and calpain interact within neutrophil membranes. Calpain inactivation by exogenous AAT remains to be investigated in more detail.

## Role of AAT Beyond Inhibition of Neutrophil Proteases

Various studies support the broad effects of AAT on neutrophil functions. For instance, AAT inhibits neutrophil superoxide production, induces IL-1Ra expression (Bucurenci et al., 1992; Tilg et al., 1993; Lewis et al., 2008) and suppresses LPS-induced IL-1β, IL-8, and TNF-α release from neutrophils (Nita et al., 2005; Al-Omari et al., 2011; Jonigk et al., 2013). Results from our group and other investigators have revealed an inhibitory effect of AAT on neutrophil chemotaxis (Nita et al., 2005; Bergin et al., 2010; Al-Omari et al., 2011; Jonigk et al., 2013) and adhesion (Carney et al., 1998; Al-Omari et al., 2011). While previous reports suggest that AAT modulates neutrophil functions via inhibition of protease activity, more recent investigations support divergent pathways involved in the actions of AAT. As an illustration, Jonigk et al. (2013) demonstrated that exogenous AAT, independent of its anti-elastase activity, can significantly lower LPS-induced release of TNF-α and KC (IL-8) in wild type or elastase-deficient mouse bone marrow neutrophils *in vitro*.

Like other proteins, AAT can function by interacting with other molecules affecting its structural, biochemical and functional properties. Growing evidence suggests that the effects of AAT on neutrophil functions might be dependent on its interaction with binding partners and on its molecular form. For example, AAT has exposed Met residues that can be easily oxidized (Li et al., 2009). Therefore, AAT can act as a direct reactive oxygen species (ROS) scavenger and modulate the levels of ROS released by neutrophils (Taggart et al., 2000). Furthermore, AAT possesses the ability to bind neutrophil alpha defensins (Panyutich et al., 1995; Spencer et al., 2004; Wencker and Brantly, 2005). The interaction between AAT and defensin is thought to play a role in regulating the ability of defensin to induce histamine release by mast cells (van Wetering et al., 1999), and to stimulate airway epithelial cells to express inflammatory cytokines (IL-8, IL-6, and MCP-1), among others (Van Wetering et al., 1997; Befus et al., 1999; van Wetering et al., 2002). In fact, Tiriveedhi et al. (2014) demonstrated that lung transplant patients who developed chronic lung allograft rejection, manifested as bronchiolitis obliterans syndrome, show increased defensin and decreased serum AAT levels along with a high concentration of AAT-defensin complexes in their bronchial lavage. These authors propose that measuring defensin and AAT levels in the serum may offer a simple, non-invasive diagnostic adjunct for chronic lung allograft rejection diagnosis (Tiriveedhi et al., 2014).

Neutrophil proteases stimulate the release of IL-8 (CXCL8) and leukotriene B4 (LTB4), potent chemoattractants of neutrophils. Recent studies by Bergin et al. (Bergin et al., 2010) and O’Dwyer et al. (O’Dwyer et al., 2015) demonstrated that AAT can directly bind IL-8 and LTB4, abrogating their chemoattractant activities. The IL-8 has been estimated to contribute about 30% of the neutrophil chemotactic activity in the sputum of emphysema patients with AATD (Woolhouse et al., 2002). Opposite to the native form of AAT, hydrophobic C-terminal fragments of AAT (Zelvyte et al., 2004a; Blaurock et al., 2016) as well as AAT polymers (Parmar et al., 2002; Mahadeva et al., 2005), were found to stimulate neutrophil chemotaxis and adhesion *in vitro* and *in vivo*. Hence, the effects of AAT on neutrophil chemotaxis seem to depend on its molecular form and operate through different pathways.

It is also important to point out that AAT interacts with low-density lipoprotein (LDL), high-density lipoprotein (HDL) (Moreno et al., 2014) and free fatty acids (Frenzel et al., 2015). In an elastase-induced pulmonary emphysema mouse model, the AAT-HDL complex showed a better protective effect in comparison to HDL or AAT alone. The protective effects of the AAT-HDL complex were characterized by a reduction in neutrophil infiltration, and declined in the concentration of pro-inflammatory cytokines (TNF-α, IL-1β, MCP-1), and MMP-2 and MMP-9 activity. In fact, HDL may transport AAT into cells (Houghton et al., 2010) where it could attenuate the deleterious effects of neutrophils.

Evidence from our group has confirmed that AAT binds with polyunsaturated fatty acids like alpha-linoleic acid and oleic acid (Frenzel et al., 2015). Dietary alpha-linolenic acid is known to affect the immune response, antioxidant status and immune-related signaling molecules. Alpha-linoleic acid (omega-3) was found to suppress neutrophil chemotactic responsiveness (Sperling, 1998).

Current studies suggest that human neutrophils are the major source of secreted IL-1β. Moreover, different authors have shown that IL-1β production by mouse or human neutrophils involves the activation of protein complexes (inflammasome) via the NLRP3/ASC/caspase-1 axis (Tamassia et al., 2012). Based on AAT being able to inhibit IL-1β release from activated neutrophils, we compared the effects of fatty acid-free and α-linoleic acid-bound forms of AAT on LPS-induced pro-IL-1β expression and active IL-1β release in purified human blood neutrophils *in vitro*. According to our results, both forms of AAT significantly reduce LPS-induced release of mature IL-1β, whereas only the fatty acid-bound form of AAT reduced both steady state mRNA levels of IL-1β and the synthesis of the IL-1β precursor. In the same LPS-stimulated neutrophils, expression of genes involved in inflammasome axis decreased significantly in the presence of fatty acid-bound AAT, but not the fatty acid-free form of AAT. Hence, the anti-inflammatory ability of AAT seems to increase due to its association with linoleic acid (Aggarwal et al., 2016).

Other studies have revealed complex formation between AAT and free heme (Cosgrove et al., 2011; Karnaukhova et al., 2012). Hemoglobin-derived free heme is a cytotoxic molecule that mediates oxidative stress, cell activation, and amplifies inflammatory responses. Our *in vitro* data provide novel evidence that AAT, as a free heme scavenger, markedly reduces or abolishes the neutrophil-activating effects of heme including neutrophil spreading, surface expression of vimentin, ROS production, and release of IL-8, and neutrophil adhesion to human endothelial cells (Janciauskiene et al., 2017). It is important to point out that the oxidized form of AAT (without anti-elastase activity), still binds heme. This implies that even if a large fraction of AAT would lose anti-elastase activity during neutrophilic inflammation, it would still be able to mediate the neutrophil-activating effects of heme. Therefore, AAT might be important protein for free heme detoxification under hemolytic conditions, especially after solid organ transplantation.

Neutrophils are becoming recognized as the regulators of the immune response by expressing cytokines, chemokines, Fc receptors and complement components for immune signaling (Mantovani et al., 2011). Emerging findings highlight the pivotal importance of AAT as a regulator of neutrophil functions, via various mechanisms.

## Neutrophil Characteristics of Individuals With Inherited AAT Deficiency

**FIGURE 3:**
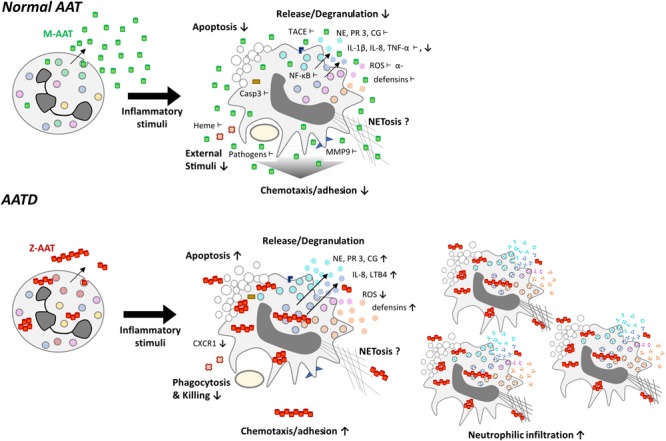
Functional differences of neutrophils expressing normal (M-variant) and deficient (Z variant) of AAT. Normal AAT is an effective anti-protease, but also possesses broad anti-inflammatory and immune-modulating properties, which are of pivotal importance in controlling neutrophil activation. Symbols show inhibitory effects of AAT on neutrophil activation: **↓** -lower levels and **⊥**-directly interacts. The AATD neutrophils producing Z variant of AAT (Glu342Lys) have an impaired ability to kill bacteria, exhibit reduced production of reactive oxygen species (ROS) and lower levels of C-X-C motif chemokine receptor 1 (CXCR1), a receptor to IL-8, which is a powerful neutrophil chemotactic factor. The Z-AAT protein forms polymers, which are chemotactic and contribute to increased neutrophil chemotaxis/adhesion and accumulation. Moreover, Z-AAT is less active as anti-protease and possesses lower immune-modulatory activities, which leads to uncontrolled neutrophil activation, and the release of pro-inflammatory molecular species.

Neutrophils are the source of AAT that is a broad regulator of neutrophil functions. For this reason, inherited and/or acquired abnormalities in the levels and/or function of AAT protein are expected to affect neutrophil properties and/or functions (**Figure 3**). Inherited AATD is a relatively common genetic condition, especially among European Caucasians, and is often undiagnosed. AATD is caused by mutations in the SERPINA1 gene and is inherited in a codominant manner (Janciauskiene and Welte, 2016). AATD related to Z (Glu342Lys) mutation is one of the most studied and clinically recognized forms. Inherited severe ZZ AATD is a cause of emphysema (loss of function due to a low serum AAT protein level, i.e., <20% of the normal 0.9–2 g/L) and liver diseases (gain of function due to the polymerisation and intracellular overload of AAT) (Zuo et al., 2016). Inherited ZZ AATD accounts for approximately 1% of cases with chronic obstructive pulmonary disease (Rahaghi et al., 2012) and is the fourth-most common reason for lung transplantation, accounting for about 6% of all adult lung transplants (Yusen et al., 2013). AAT deficiency is also associated with an increased prevalence of bronchiectasis, asthma, neutrophilic panniculitis, and Wegener’s granulomatosis. Recent study have suggested an association between SERPINA1 gene polymorphism, levels of AAT and sickle cell disease (Carvalho et al., 2017).

Z-AATD does not occur due to a lack of AAT protein biosynthesis, but from Z-AAT polymerisation and intracellular accumulation (Janciauskiene and Welte, 2016). Other investigators have demonstrated that plasma from Z-AATD individuals contains significant amounts of circulating AAT polymers (Janciauskiene et al., 2002; Tan et al., 2014). These extracellular polymers are pro-inflammatory, and chemotactic for neutrophils (Parmar et al., 2002). This latter, together with impaired anti-serine protease activity of Z-AAT, may enhance susceptibility to the development of emphysema (Clemmensen et al., 2011).

It is well-documented that homozygous and heterozygous individuals for the Z allele of AAT, have increased neutrophil influx into the airways (Malerba et al., 2006). This increased neutrophil accumulation is thought to be linked to: (i) unopposed neutrophil proteases that can enhance production of LTB4 and IL-8 thereby enhancing neutrophil recruitment into the lung (Devaney et al., 2003) and/or (ii) lack of AAT to bind and neutralize excessive release of defensins, IL-8, LTB4, soluble immune complexes (Bergin et al., 2010) and other putative substances that enhance neutrophil recruitment. As mentioned above, Z-AAT polymers *per se* can act as neutrophil chemoattractants (Mulgrew et al., 2004). Likewise, AAT polymer induced chemotaxis and IL-8 release may act synergistically (Persson et al., 2006). Interestingly, AAT polymers have been suggested to act as a template for the binding of bacterial or yeast products leading to the generation of pro-inflammatory and chemotactic forms of the polymer (Persson et al., 2006).

Various studies have demonstrated that AATD neutrophils have an impaired ability to kill bacteria, such as *Pseudomonas aeruginosa* and exhibits reduced production of ROS (McCarthy et al., 2016). Reduced levels and functional activity of Z-AAT lead to the shift of equilibrium in favor of proteases. Hence, one mechanism by which impaired ability of AATD neutrophils to kill bacteria might occur is through NE-mediated inactivation of CXCR1. Decreased neutrophil surface expression of CXCR1 impairs the respiratory burst and has been shown to correlate with reduced neutrophil bactericidal capacity in patients with chronic pulmonary disease (Hartl et al., 2007).

The AATD neutrophils are also dysfunctional due to accumulation of the misfolded Z-AAT protein in the endoplasmic reticulum (ER) of neutrophils, leading to ER stress and accelerated neutrophil apoptosis, which may in turn contribute to increased susceptibility to infection (Hurley et al., 2014).

Alpha1-Antitrypsin also seems to play a role in TNF-α expression and activity. TNF-α self-regulates its own gene expression as well as that of other inflammatory cytokines (Spriggs et al., 1990; Sato et al., 1996; Fiedler et al., 1998). AAT downregulates TNF-α gene expression by inhibiting the nuclear factor kappa-light-chain-enhancer of activated B cells (NF-κB) signaling (Bergin et al., 2014). In neutrophils isolated from Z-AAT individuals, this mechanism seems to be impaired. Consequently, Z-AAT neutrophils show increased degranulation, which may lead to the development of autoantibodies, specifically against lactoferrin. Anti-lactoferrin antibodies are associated with increased ROS and neutrophilic inflammation in various diseases, like rheumatoid arthritis (Chikazawa et al., 2000), systemic lupus erythematous (Caccavo et al., 2005), and inflammatory bowel disease (Roozendaal et al., 1998). These elevated anti-lactoferrin autoantibodies in AATD highlight the contribution of the neutrophils to the inflammatory burden.

Stimulated neutrophils produce neutrophil extracellular traps (NETs), composed of chromatin fibers with attached several proteins; including histones and components of primary and secondary granules (like NE and PR3). NET activation and release (NETosis), is a dynamic process that begins with the activation of peptidylarginine deiminase 4 (PAD4), which then leads to histone citrullination, chromatin decondensation, and the nuclear localization of granular enzymes (e.g., NE). NETosis can be suicidal (NETs release characterized by nuclear swelling, and membrane rupture) and vital (NETs release without loss of nuclear or plasma membrane) forms (Delgado-Rizo et al., 2017). NET formation is strongly dependent on ROS production (Soehnlein et al., 2017). NETs can capture and kill gram-positive and -negative bacteria, and fungi. However, in a chronic setting NETs might also propagate unwanted inflammatory reactions. For example, increased NETosis and NET-associated components (NE, PR3, lipocalin, and others) have be linked with delayed wound healing in diabetes (Fadini et al., 2016). In patients with type 1 diabetes, elevated formation of NETs was associated with increased NE and PR3, and decreased circulating levels of AAT (Wang et al., 2014).

As mentioned above, AAT not only inhibits NE and PR3 but also suppresses the generation of ROS required for NET formation. Thus, hypothetically neutrophils from Z-AATD patients must be more prone to form NETs, whereas one would expect exogenous AAT to inhibit this process. To address this paradox, we employed an *ex vivo* model and used the well-characterized inducer of NETs, phorbol myristate acetate (PMA). We obtained blood neutrophils from ZZ AATD-related emphysema patient before and directly after therapy with AAT, and incubated with PMA for 4 h *in vitro*. Our findings provided evidence that AAT does not inhibit PMA-induced NET formation, but induces remarkable NET shape changes and reduces NET adhesion. Moreover, we found that AAT localized in the NETs structures, although separately from NE (Frenzel et al., 2012).

Clearly, additional investigations are required to examine more closely the effect of native and polymeric Z-AAT on neutrophil NET formation.

## The Role of AAT/Neutrophil Interplay in Tumorigenesis and Metastasis

Elevated counts of blood neutrophils is a strong predictor of poor clinical outcomes among patients with diverse cancer types (Jensen et al., 2009; Ilie et al., 2011; Li et al., 2011; Rao et al., 2012; Donskov, 2013; Shen et al., 2014; Coffelt et al., 2016). Neutrophils are also present in the tumor microenvironment and referred to as tumor-associated neutrophils (TAN). Experimental studies have shown that chemokines and cytokines, such as IL-8, IL-1β, IL-6, and TNF-α, produced by the cells present in the tumor microenvironment as well as by the tumor on its own, can positively influence neutrophil recruitment (Waugh and Wilson, 2008; Lechner et al., 2010) and thereby promote angiogenesis, tumor progression, and metastasis (Bekes et al., 2011). Most of the neutrophil-induced pro-tumor effects are associated with the release of NE, CG and PR3, which activate MMPs, and thus mediate tumor invasiveness via their degrading activity against extracellular matrix proteins. In line with this, preclinical studies revealed that genetic deletion or pharmacological inhibition of NE reduces tumor burden and metastatic potential (Sun and Yang, 2004; Ghaedi et al., 2011; Lerman and Hammes, 2017).

In view of the pro-tumorigenic effects of neutrophil proteases, it is likely that AAT can counteract tumor progression and metastasis. Indeed, Huang et al. have shown that systemic administration of AAT delays tumor progression and reduces tumor capillary density in nude mice (Huang et al., 2004). Another study found that curcumin, a polyphenolic natural product, inhibits NE-induced tumor proliferation by upregulating the expression of AAT (Xu et al., 2012). In recent years, multiple studies have shown links between AAT levels and cancer prognosis, but the conclusions are inconsistent. It has become increasingly apparent that cancer patients with high plasma levels of AAT have worse prognosis (Higashiyama et al., 1992; Zelvyte et al., 2004b; Li et al., 2011) although there is no evidence to point to the specific pro-tumor effects of AAT. Interestingly, our *in vitro* studies showed that AAT, depending on its molecular form, could either promote or inhibit effects of neutrophils on cancer cell proliferation and invasiveness (Zelvyte et al., 2004a). As discussed earlier, *in vivo* AAT is found in native, inhibitory active form, but also in modified forms, including oxidized, complexed with other molecules and polymerized. Native AAT, acting as an inhibitor of NE and PR3, may protect extracellular tissues from degradation and may also inhibit tumor progression, while modified, non-inhibitory forms of AAT may favor tumor growth and progression. For example, Kataoka et al. (1999) found that the C-terminal fragment of AAT generated during the cleavage with the MMPs contributes to tumor progression *in vivo*.

Some of the above-mentioned contradictory results perhaps we can explain if we take into account the phenotypic diversity of neutrophils. Neutrophils exhibit substantial plasticity and in response to the microenvironment can be polarized to an N1 (anti-tumor) or N2 (pro-tumor) phenotype (Fridlender et al., 2009). For example, N1 neutrophils produce higher levels of TNFα, NO, and ROS, and are able to kill cancer cells whereas N2 neutrophils express high levels of C-X-C chemokine receptor type 4 (CXCR4 or CD184), gelatinase B/MMP9, among other markers, and act as mediators of tumor progression (Fridlender et al., 2009). Thus, hypothetically shifting these N2 neutrophils to N1 phenotype or *vice versa* by AAT would significantly inhibit or enhance tumorigenesis. Understanding how neutrophils polarize toward pro- or anti-tumor phenotype, and if and how AAT can affect this switch, will be crucial for explaining the AAT/neutrophil interplay in tumorigenesis.

## The State of Clinical Development of AAT Drugs

The augmentation therapy with pooled human plasma AAT was introduced in 1988 based on biochemical data showing that intravenous infusions of AAT neutralize NE activity within the lungs of emphysema patients with inherited AATD (Wewers et al., 1987). Cystic fibrosis (CF) is another hereditary lung disease associated with a neutrophil-dominated inflammation. Although the levels of AAT might be normal in this condition, the NE burden maybe so large that it overwhelms the protection provided by the AAT. Therefore, AAT can be an attractive therapeutic option for CF patients (McElvaney, 2016). In fact, during the years AAT drug demonstrated encouraging impact on the inflammatory processes in the lungs of AATD-related emphysema as well as in patients with CF.

The vast body of published pre-clinical studies on the efficacy of AAT therapy provide bases for expanding the use of AAT outside the context of inherited AATD. Specifically, AAT may have beneficial effects in diseases characterized by the neutrophilic-inflammation like asthma, bronchiectasis, panniculitis, inflammatory bowel diseases, rheumatoid arthritis, ischemic heart disease, cancer and others (Lewis, 2012; Janciauskiene and Welte, 2016). In parallel, new types of AAT, new routes of administration and the physiologic, radiologic, and clinical readouts for AAT therapy are under development. These include the production of recombinant AAT and development of inhaled preparations of AAT (Monk et al., 2013). An alternative strategy uses non-viral gene transfer, gamma-retrovirus, recombinant adenovirus (rAd), and recombinant adeno-associated virus (rAAV) vectors to express AAT. The challenge is to achieve long-term expression of large quantities of AAT (Mueller and Flotte, 2013). Moreover, scientists consider AAT protein structure-based drug design approaches and strategies preventing pathogenic conformational changes of AAT. Available crystallographic datasets for wild type and mutant AAT proteins reveal considerable variability in the surface clefts and suggest AAT protein as a good target for structure-based drug design. In parallel, a number of promising approaches for the prevention of pathogenic polymerization and maintenance of the AAT protein in an active conformation are under development; these include chemical chaperones that stabilize the AAT protein and peptides that block the polymerization of AAT. None has yet been approved for use in man (Chang et al., 2011; Nyon and Gooptu, 2014).

Finally, there is some data to indicate that AAT functions not only as a protease inhibitor but also as anti-angiogenic factor. Researchers evaluated the synthesis and release of AAT by lentiviral transduction in human mesenchymal stem cells (hMSCs)-derived from bone marrow donors. Results from this study suggest a potential role of AAT as an inhibitor of angiogenesis when AAT-hMSCs home to a tumor site (Ghaedi et al., 2011).

## Conclusion

The accumulation of neutrophils and secretion of active serine proteases are hallmarks of numerous inflammatory conditions. Qualitative and /or quantitative defects of AAT, a vital regulator of neutrophil functions, may have significant implications in the development of chronic inflammation and diseases. It is important to note that many of the effects of AAT on neutrophil behavior operate through different pathways, some through protease inhibition, others through hydrophobic interactions between AAT and other molecules, or through direct AAT binding to specific receptors. Therefore, it remains of critical importance to characterize and understand the mechanisms involved in AAT biological activities, and to relate these findings to clinic practice. A more detailed understanding of the factors that influence the functional properties of AAT may offer novel prospects for understanding interplay between AAT and neutrophils in carriers of normal and defective variants of AAT.

## Author Contributions

SJ, SW, SI, BO, TG, TW, and JC-W participated in conceptual work, figure preparation and writing of the manuscript, and approved it for publication.

## Conflict of Interest Statement

The authors declare that the research was conducted in the absence of any commercial or financial relationships that could be construed as a potential conflict of interest. The reviewer MD and PB and handling Editor declared their shared affiliation.
